# Treatment of Arrow Wounds: A Review

**DOI:** 10.7759/cureus.2473

**Published:** 2018-04-13

**Authors:** Rafik Shereen, Rod J Oskouian, Marios Loukas, R. Shane Tubbs

**Affiliations:** 1 Department of Anatomical Sciences, St. George's University School of Medicine, St. George, GRD; 2 Neurosurgery, Swedish Neuroscience Institute; 3 Neurosurgery, Seattle Science Foundation

**Keywords:** management, arrowhead, trauma, penetrating injury

## Abstract

The arrow is one of the oldest weapons invented that has ties back to ancient civilization. With the advancement of modern weaponry, literature concerning the management of this traumatic wound has dwindled.  However, there are older written accounts that have led to our understanding of how we manage injuries inflicted by the arrow. One of the more comprehensive accounts was produced in the 19th century by a United States (US) Lieutenant Colonel named JH Bill. Recent cases in forensic pathology, as well as instances of trauma concerning arrowhead injuries, have documented a lack of comprehensive literature for the management of such injuries. Thus, the goal of our review is to evaluate the literature and provide a record of the different presentations, complications, and ways to manage arrow injuries.

## Introduction and background

It has been estimated that the arrow has killed more individuals than any other weapon in history. However, many physicians and surgeons today regard this weapon as extinct as a cause of traumatic injuries [1]. Nonetheless, the historical significance of the bow and arrow has been cited by many as a turning point in medicine. The arrow was a deadly weapon in its most basic form; however, ancient civilizations developed multiple additions to enhance its effectiveness [2]. Barbs, metal fragments, and even crescent-shaped arrowheads have been recorded throughout history [2]. These variations proved difficult to manage, eventually leading to the development of certain tools specific to the extraction of arrows. Karger et al. argued that it was the advancements in developing tools and approaches for the extraction of arrows that served as a major stimulus for modern surgery [1].

In their report, Karger et al. cited the ancient Greek, Celsus, as the first to devote an entire chapter to arrow wounds and treatment of these wounds with surgical techniques [2]. These authors went on to note that, even at this early stage, Celsus understood the importance of pushing an arrow through emergence as opposed to pulling with traction. Celsus would eventually develop a surgical instrument, which he called the spoon of Diocles, to aid in this endeavor [2]. The story of Machaon, wounded by an arrow of Paris, was discussed at length in the Iliad by Homer [2]. Homer even goes on to describe the poor surgical approaches that were suffered by Machaon in attempts to remove these arrows [2]. Since then, many cultures have documented a great amount of knowledge about arrow wounds and their treatment.

At the turn of the 20th century, the occurrence of arrowhead injuries declined, and with it, so too did the literature concerning their management [2-3]. Recently, however, there has been a resurgence of documented cases concerning the difficulty in managing such traumatic injuries [3]. In 2010, a case report was published by Paramhans et al. describing a 35-year old male who presented with an impacted metallic arrowhead in his brainstem [3]. Their report concluded with an improvement of the patient’s neurological deficits after removal of the arrowhead.  However, Paramhans et al. noted that one of the most difficult aspects of the penetrating injury was the removal of the arrow. The base of the arrow was located outside of the skull, disabling approaches via craniotomy. The tip was located near the brainstem, making extraction even more dangerous [3]. Paramhans et al. went on to note that while a fair amount of literature exists on trauma wounds involving guns and knives, there is very little research with respect to penetrating, non-gunshot projectile injuries [3]. In addition, a recent poll by Responsive Management for the Archery Trade Association conducted in 2014 revealed an increase in the participation in archery recreational events amongst United States (US) adults [4]. A more recent experiment conducted by forensic pathologists demonstrated how modern arrowheads can mimic both stab and gunshot wounds [5]. This experiment also noted that studies on arrow injuries were limited, and while they supplied a detailed report for classification of morphologic variations, they failed to mention management or treatment options for an arrow wound [5].

One of the last extensive works concerning the management of arrow wounds was an article published in the 19th century by JH Bill, who served as the Surgeon and Brevet Lieutenant-Colonel in the United States Army. His research concerning traumatic injuries inflicted by arrows is a comprehensive account, which has been included as a focus in this review.

## Review

Presentation of an arrow wound

According to Bill, an arrow causes a wound that is both punctured and incised [2]. This wound often allows the outward flow of discharges due to its structure, high velocity, and the fact that an arrow wounds tissue in a continuous fashion. As a result, arrow wounds that did not pierce regions in which microflora could be released have rarely resulted in infections. Arrows also remain in their target and, in such instances, serve as a tamponade to blood vessels and internal luminal structures. If the arrow had completely pierced an individual, it would leave distinct entry and exit wounds. The entry wound resembles that of a bullet (a slit that is darkened, bruised, and depressed), and the exit wound looks like a simple slit. If only one of the wounds is found, it can be mistakenly assumed as a bullet or stab wound. In addition, an arrow can tangentially hit its target, resulting in a linear slit that resembles a laceration. It is interesting to note that these same findings were discovered by Sung et al. while discussing the forensic pathology of arrow wounds in the 21st century [5].

In his account, Bill gathered more than 150 cases of arrow injuries to evaluate which regions of the body are more liable to be wounded, as well as which wounds are more likely to result in death [2]. The regions were divided into head and neck, thorax, heart, abdomen, and upper and lower extremities. Twenty-six of the 154 cases were injured in the head and neck, 28 were injured in the thorax, two were injured in the heart directly, 34 suffered abdominal injuries, 46 suffered injuries in the upper extremities (the most injured region of the body), and of the cases, 18 had a lower extremity injury. Most of the causes of death by arrows were secondary to infection, with peritonitis following an arrow that pierces the abdominal cavity being the most common cause of death. Second to peritonitis, the individual may die due to massive hemorrhage. Other causes of death included pneumonia, encephalitis, compression of the brainstem, empyema, tetanus, and shock.

Treatment

General

For arrows that passed completely through, Bill endorsed an assessment of the injured body part for any loss of function or damage to major organs [2]. If no such trauma was observed, the wound required minimal intervention.  Bill proclaimed that the cleanness of the cut made by an arrow allowed for a healing of the wound to occur without suppuration. In areas that were in a dry climate, a wound could heal via primary intention in as quickly as two days [2].

For intact arrows that were lodged, the most important thing to determine was if the arrow had impacted bone. The 19th-century physician may have determined this by gently twisting the arrow at its tip [2]. The slightest mobility indicated an arrow that was free of impaction. In more modern case reports, imaging modalities, such as X-ray and computed tomographic (CT) scans, were used to determine if the arrow had impacted bone. Once it was determined that the arrowhead was free of bone, the next step in management was to decide whether the arrow should be pulled out or pushed through. This was dependent on the depth of the arrow, as well as the tissues it may encounter. When it was decided that the arrow could successfully be removed by emergence, the shaft was to be lubricated and firmly pushed through at its base. As the tip of the arrowhead became visible underneath the skin, a scalpel would be used to release the arrow from the integument, ensuring no part breaks or was left behind. Once it was confirmed that the entire arrowhead had been removed, the rest of the arrow could be pushed through. If any portion of the arrow was suspected to remain in the wound, a drainage tube would be used to prevent suppuration.

If it was not feasible to push an arrow through, then it was extracted, which was done by grasping the arrowhead. However, the shaft of the arrow tended to be tightly surrounded by the skin, which proved to be problematic. Thus, for arrows that were more superficial, it was recommended to make an incision allowing a probe to visualize or feel for the arrowhead and eventually dressing forceps to retrieve it. The arrowhead and shaft were removed at the same time, grasping each together, so as to not let the arrowhead break from the shaft. If the arrow was deeply lodged, the 19th-century physician had to be more creative. Many tools have been developed for this very purpose, and in his report, Bill accounted for the more popular surgical instruments used for extraction of arrows at the time. His figures have been included in this review [2].

**Figure 1:**
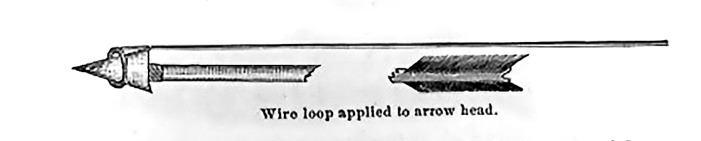
Extraction of non-impacted arrowheads Figure copied from: Bill JH: Sabre and bayonet wounds; arrow wounds. International Encyclopedia of Surgery: A Systematic Treatise on the Theory and Practice of Surgery By Authors of Various Nations. Ashhurst J (ed): William Wood & Co, New York; 1882. 2:101-117

Figure 1 was an instrument intended for removing arrows that were deeply lodged but not impacting bone [2]. The tip of the probe was a loop about an inch in diameter. The rest of the instrument was made from a stout wire that was longer than the arrowhead.  The instrument was intended to probe for the tip of the arrowhead and then hook around it, securing the arrowhead as the physician pulled back on the shaft of the arrow and the probe at the same time, securely removing the whole arrow at once. As depicted in the figure the protruding end of the arrow was often broken off [2].

**Figure 2:**
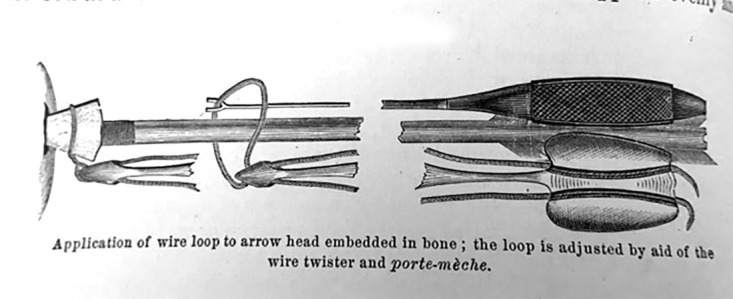
For arrowheads embedded in bone Figure copied from: Bill JH: Sabre and bayonet wounds; arrow wounds. International Encyclopedia of Surgery: A Systematic Treatise on the Theory and Practice of Surgery By Authors of Various Nations. Ashhurst J (ed): William Wood & Co, New York; 1882. 2:101-117

Figure 2 was an instrument developed for the extraction of arrowheads that had impacted bone [2]. Because of the considerable force required to extract an arrowhead impacted in bone, the shaft was often broken. The inability to loop a wire over the tip of the arrow proved to be more problematic. To overcome this obstacle, a loop was passed down the shaft of the arrow, larger than the arrowhead itself, until it was positioned over the arrowhead. The end of the loop was then threaded through a device similar to a Coghill suture twister. The wires were drawn tighter and then the loop firmly attached to the arrowhead; due to the shape of the arrowhead, it would have only strengthened its hold when traction was applied.

**Figure 3:**
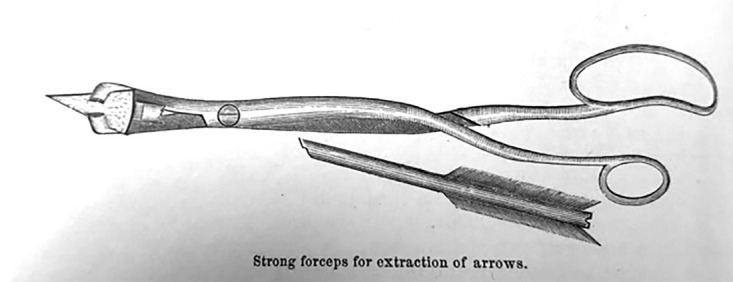
Bill's extraction tool for arrowheads impacted in bone Figure copied from: Bill JH: Sabre and bayonet wounds; arrow wounds. International Encyclopedia of Surgery: A Systematic Treatise on the Theory and Practice of Surgery By Authors of Various Nations. Ashhurst J (ed): William Wood & Co, New York; 1882. 2:101-117

Figure 3 represents another instrument used for extraction of arrows impacted in bone that Bill devised [2]. He developed forceps with jaws that were flat and bent at right angles, similar to a dentist’s forceps, intended for providing maximum torque with minimal bending or breaking. The jaws were designed as an elliptical loop, acting like a sheath of a knife blade over the arrowhead to aid in its extraction. 

**Figure 4:**
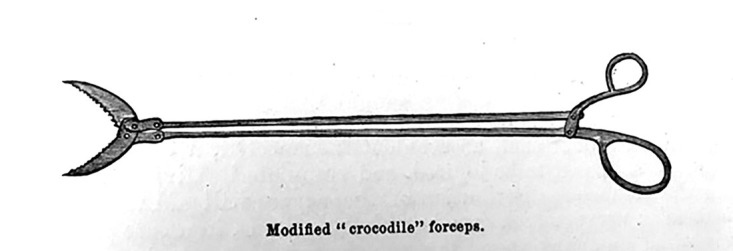
Crocodile forceps for fragmented arrowheads Figure copied from: Bill JH: Sabre and bayonet wounds; arrow wounds. International Encyclopedia of Surgery: A Systematic Treatise on the Theory and Practice of Surgery By Authors of Various Nations. Ashhurst J (ed): William Wood & Co, New York; 1882. 2:101-117

While the arrow was less likely to break into fragments than a bullet, if it did, fragments would be removed with “crocodile” forceps as depicted in Figure 4 [2]. Again, in his account, Bill was quick to recommend exploration prior to removal of the arrow to ensure any possible fragments that broke off were removed.

Treatment for Specific Injuries

**Figure 5:**
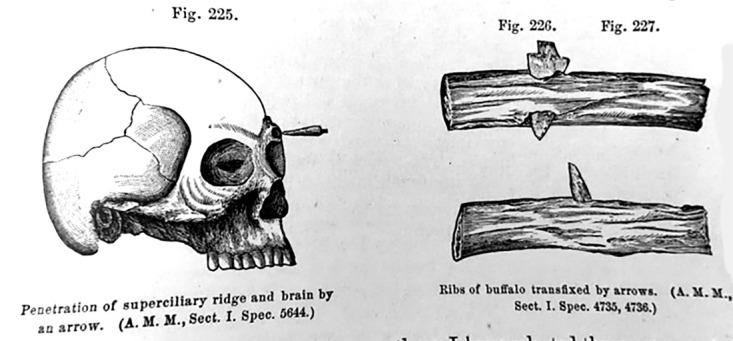
Arrow impacting the superciliary ridge (left). On the right are images of an arrowhead impacted in rib cages of a buffalo. Figure copied from: Bill JH: Sabre and bayonet wounds; arrow wounds. International Encyclopedia of Surgery: A Systematic Treatise on the Theory and Practice of Surgery By Authors of Various Nations. Ashhurst J (ed): William Wood & Co, New York; 1882. 2:101-117

**Figure 6:**
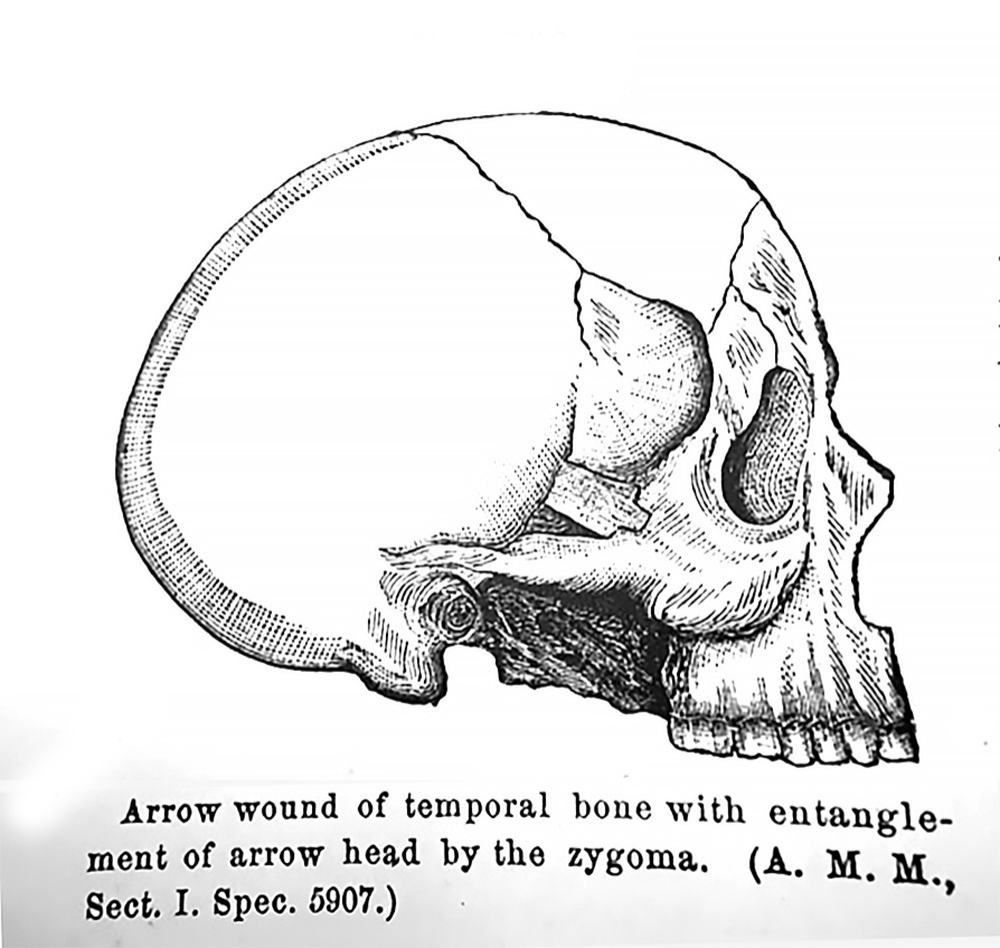
Arrowhead lodged in the zygomatic arch. Figure copied from: Bill JH: Sabre and bayonet wounds; arrow wounds. International Encyclopedia of Surgery: A Systematic Treatise on the Theory and Practice of Surgery By Authors of Various Nations. Ashhurst J (ed): William Wood & Co, New York; 1882. 2:101-117

In most cases, especially if the arrow remained lodged in the individual, injured vessels would rarely hemorrhage. Larger vessels were more susceptible to hemorrhage, and if they did, the injury was explored and the bleeding point addressed with a ligature. The treatment for arrow wounds of joints typically resulted in good outcomes. Arrows can be shot with a powerful enough momentum that if it hit its target at the correct angle it could penetrate the calvaria [2]. The extent of the injury was dependent on the location and the depth of the arrow, some obviously resulting in immediate death. In his report, Bill described an example of an arrow piercing the thickest part of the superciliary ridge, as shown in Figure 5, which he found at the now demolished Army Medical Museum. In his account, Bill noted that the individual did not report any symptoms. If, upon removal of the arrow, there is suspicion of hemorrhage, trephination of the skull would be performed and the bleeding point addressed with lint cloth or ligature if possible. Arrow wounds of the face often resulted in considerable hemorrhage. These wounds were also considered dangerous due to the sponginess of facial bones. As a result, the arrowhead could again lodge itself in a manner that proved to be problematic for the surgeon. In another specimen of which Bill provides, a representative figure (Figure 6), an arrow, finds itself lodged under the zygomatic arch of the skull and embedded in the temporal muscles of the face. Here again, Bill recommended exploration to observe the nature of which the arrowhead had found itself lodged.

 

Arrow wounds to the thorax had to be evaluated carefully for penetration into the lung, in which case the individual would die 72% of the time [2]. Unlike a bullet wound, arrow wounds rarely resulted in the collapse of the lung, as the arrow usually remained lodged within the thorax. However, if the lung were to collapse, the risk of hemorrhaging was decreased, as a collapsed lung served to compress vessels. If the patient survived the period of hemorrhage, then the prognosis was favorable. However, if the arrowhead found itself lodged in lung tissue, the prognosis was less favorable; often the arrowhead would have to be left in the lung, ultimately resulting in the demise of the individual. The same rules that applied to arrows passing through in the body in general applied to an arrow passing through the thorax, the exception of which was with respect to arrows that penetrated the rib. In that case, the physician would recommend trephination at the point of greatest impact. All instances where the arrow either pierced both lungs or the heart resulted in death.

Arrow wounds of the abdomen and pelvis were generally fatal in the 19th century. While peritonitis and fecal extravasation often led to fatal infections, the other main cause of death for abdominal wounds was hemorrhage [2]. An interesting case presented when a native American was found to have a large renal calculus resulting from an iron arrowhead that had found itself at the base of the bladder. Treatment of an arrow wound of the abdomen was dependent on the type of arrow, controlling the hemorrhaging, suturing any major vessels or intestines that were injured, thoroughly cleansing any extravasated fecal matter, and rest. Once these were addressed, as they were at the time, the abdomen would be closed with a figure of eight sutures passing through the muscles, as well as through the integument. It should be noted that the results of his study were limited by the lack of current advancements in medicine.  

Bill stressed the importance of exploration prior to removal, giving great care to the removal of the shaft and arrowhead as one piece, and extensive incisions along the direct path of the arrow to aid in its removal as a whole. Though his work was limited by the era in which it was conducted, Bill was able to provide documentation for different techniques of managing the extraction and removal of arrowheads.

## Conclusions

Due to their unique method of penetration and relative complexity for removal, the removal of an arrow from the individual it impales has given rise to multiple tools and techniques that certain aspects of modern surgery can be attributed to. While the initial practical application of the arrow has been, for the most part, replaced by the firearm, it is still in use today, and there are cases that have highlighted the limited research in dealing with such a traumatic injury. Forensic pathologists have noted that the periodic use of arrows, both modern and historical, in homicide cases have pointed out the lack of literature concerning the appearance of these wounds. In addition, there lacks any major source for the current management of arrowhead wounds. Given the recent increase in archery participation, as well as the cyclical finding of arrowhead injuries, this review provides proposed techniques for the management of arrowhead injuries which may not have been previously considered.
